# Intrastriatal Administration of AAV5-miHTT in Non-Human Primates and Rats Is Well Tolerated and Results in miHTT Transgene Expression in Key Areas of Huntington Disease Pathology

**DOI:** 10.3390/brainsci11020129

**Published:** 2021-01-20

**Authors:** Elisabeth A. Spronck, Astrid Vallès, Margit H. Lampen, Paula S. Montenegro-Miranda, Sonay Keskin, Liesbeth Heijink, Melvin M. Evers, Harald Petry, Sander J. van Deventer, Pavlina Konstantinova, Martin de Haan

**Affiliations:** 1 uniQure biopharma B.V., 1105 BP Amsterdam, The Netherlands; a.vallessanchez@uniqure.com (A.V.); m.lampen@uniqure.com (M.H.L.); p.miranda@uniqure.com (P.S.M.-M.); s.keskin@uniqure.com (S.K.); l.heijink@uniqure.com (L.H.); m.evers@uniqure.com (M.M.E.); haraldpetry01@aol.com (H.P.); Pavlina.konstantinova@vectorytx.com (P.K.); 2 Department of Gastroenterology and Hepatology, Leiden University Medical Center, 2333 ZA Leiden, The Netherlands; Sander.van.deventer@forbion.com; 3 Madeha Management & Consultancy, 1222 LM Nederhorst den Berg, The Netherlands; madeha.mc@gmail.com

**Keywords:** AAV5, Huntington disease, miRNA, gene therapy

## Abstract

Huntington disease (HD) is a fatal, neurodegenerative genetic disorder with aggregation of mutant Huntingtin protein (mutHTT) in the brain as a key pathological mechanism. There are currently no disease modifying therapies for HD; however, *HTT*-lowering therapies hold promise. Recombinant adeno-associated virus serotype 5 expressing a microRNA that targets *HTT* mRNA (AAV5-miHTT) is in development for the treatment of HD with promising results in rodent and minipig HD models. To support a clinical trial, toxicity studies were performed in non-human primates (NHP, *Macaca fascicularis*) and Sprague-Dawley rats to evaluate the safety of AAV5-miHTT, the neurosurgical administration procedure, vector delivery and expression of the mi*HTT* transgene during a 6-month observation period. For accurate delivery of AAV5-miHTT to the striatum, real-time magnetic resonance imaging (MRI) with convection-enhanced delivery (CED) was used in NHP. Catheters were successfully implanted in 24 NHP, without neurological symptoms, and resulted in tracer signal in the target areas. Widespread vector DNA and mi*HTT* transgene distribution in the brain was found, particularly in areas associated with HD pathology. Intrastriatal administration of AAV5-miHTT was well tolerated with no clinically relevant changes in either species. These studies demonstrate the excellent safety profile of AAV5-miHTT, the reproducibility and tolerability of intrastriatal administration, and the delivery of AAV5-miHTT to the brain, which support the transition of AAV5-miHTT into clinical studies.

## 1. Introduction

Huntington disease is a rare, fatal, neurodegenerative genetic disorder that affects motor function and leads to behavioral symptoms and cognitive decline, resulting in total physical and mental deterioration [1]. HD is caused by the expansion of CAG trinucleotide repeats in exon 1 of a multifunctional gene coding for a protein called Huntingtin [2]. Mutant HTT protein aggregation and RNA toxicity [3] lead to progressive striatal and cortical atrophy observable by MRI [4,5].

There are currently no disease-modifying treatments for HD, although strategies targeting *HTT* hold great promise [6]. These include antisense oligonucleotides (ASO), small molecules and gene therapy approaches [7,8]. In this regard, results of a phase 1–2a study of an antisense oligonucleotide (ASO) targeting the HTT protein have been published recently, which are suggestive of target engagement [9,10]. The intrastriatal administration of gene therapy with a recombinant adeno-associated virus serotype 5 expressing a microRNA (miRNA) targeting *HTT* (AAV5-miHTT) also holds promise for lowering mRNA and HTT protein in the brain and slowing HD progression [6,7].

There are crucial differences between targeting the HTT protein with ASO and with AAV-miRNA. ASOs are usually administered via intrathecal infusion, whereas AAV vectors containing a DNA construct encoding for miRNA transgenes are generally infused directly into relevant target areas in the brain [7,11]. Additionally, ASOs offer short-term HTT lowering, so regular re-administration is required, while AAV vectors such as AAV5-miHTT can achieve long-term HTT lowering with a single treatment [7,11,12]. There are also differences in the brain distribution of ASO and AAV vectors following the different injection routes. Intrathecal ASO administration favors HTT lowering in the cortex but is less effective in the striatum, and most likely does not reach the putamen sufficiently, which is the primary site of HD pathology [11,13]. In contrast, intrastriatal administration of AAV5-miHTT results in widespread distribution of mi*HTT*, Huntingtin mRNA and HTT protein lowering in most brain areas including the striatum and cortex [7].

Preclinical data in a variety of transgenic HD animal models show that intrastriatal administration of AAV5-miHTT leads to widespread transduction across the entire central nervous system [14], as previously shown for AAV5-green fluorescent protein (GFP) in non-human primates [15]. Vector DNA and mi*HTT* expression was shown to be sustained [12,14,16,17] and demonstrated strong lowering of mutHTT mRNA and mutHTT protein [12,14,17]. The direct intraparenchymal administration of AAV5-miHTT also suppresses mutHTT protein aggregation and prevents neuronal dysfunction in rats [16], improves phenotypic features such as motor coordination, behavior and cognition, reverses striatal volume loss, and increases survival [14,16,17].

AAV5-miHTT is a promising gene therapy candidate to treat HD in humans given the strength of preclinical data in small and large animals. The objectives of these toxicity studies in Sprague-Dawley (SD) rats and NHP (*Macaca fascicularis)*, conducted according to Good Laboratory Practice (GLP) principles, were to examine tolerability of the administration procedure into the striatum and the safety and biodistribution of AAV5-miHTT during a 6-month observation period, to support the transition of AAV5-miHTT into clinical studies. Since the mi*HTT* binding site does not completely match the NHP or rat *HTT* mRNA, the safety of *HTT* lowering was outside of the scope of the safety evaluation in these studies. The tolerability and reproducibility of real-time MRI guided intrastriatal convection enhanced delivery of AAV5-miHTT was evaluated in the NHP study. *Macaca fascicularis* was selected as the most relevant species because of the similarities between the brains of NHP and humans [18]. In addition, primates are natural hosts of AAV, and are therefore recommended for safety studies investigating the use of AAV vectors for gene therapy [19]. Rats were included to facilitate multiple interim timepoints during the 6-month observation period, for the evaluation of biodistribution and safety over time.

## 2. Materials and Methods

### 2.1. Ethical Statement

All studies were performed in compliance with GLP regulations at contract research organizations (CROs) accredited by the Association for Assessment and Accreditation of Laboratory Animal Care (AAALAC). In the NHP study conducted by Covance Preclinical Services (Münster, Germany) all procedures complied with the German Animal Welfare Act and were approved by the local Institutional Animal Care and Use Committee (Approval ID: 84-02.04.2017.A183, 07 September 2017, Landesamt für Natur, Umwelt und Verbraucherschutz Nordrhein-Westfalen). The study in SD rats, accommodated by Envigo CRS Limited (now Covance Laboratories Ltd., Huntingdon, United Kingdom), was conducted in accordance with the applicable sections of the United Kingdom Animals Act 1986 and Amendment Regulations 2012 and was approved by the Animal Welfare and Ethical Review Body (AWERB) of Covance Laboratories Ltd. (Approval ID: PE084706C, 29 September 2017).

### 2.2. Study Design and Animals

#### 2.2.1. NHP

Cynomolgus macaques (*Macaca fascicularis*) of Asian origin (Vietnam) aged 2.6–3.5 years, were randomized to a single bilateral intrastriatal administration of control or AAV5-miHTT at 2 × 10^12^, 7 × 10^12^ or 2 × 10^13^ genome copies (gc)/animal. Each group included 3 males and 3 females resulting in 24 NHP. The study was conducted at Covance Preclinical Services in Munster, Germany. NHP with an anti-AAV5 neutralizing antibody (NAb) titer inhibitory concentration (IC) 50s < 100 were selected for the study. Animals of the same sex and dose group were housed in group cages in a climate-controlled room, with a minimum of eight air changes/hour and a 12-h light 12-h dark cycle. The temperature and relative humidity ranges were 19–25 °C and 40%–70%, respectively. Tap water was available ad libitum. Twice daily, the animals were offered a certified lab diet (LabDiet 5048). Fresh fruit and vegetables were provided regularly as a food supplement. Animals were inspected at least twice daily for evidence of ill-health or a reaction to treatment. A detailed weekly physical and neurological examination was performed to monitor general health.

#### 2.2.2. Rats

SD rats (*Rattus norvegicus*, Crl:CD(SD)), aged 8–11 weeks, were randomized to the control group, or AAV5-miHTT at 4 × 10^9^, 4 × 10^10^, 4 × 10^11^ gc/animal. Male and female SD rats were supplied by Charles River (UK) Ltd. and the study was conducted at Envigo CRS Limited, Huntingdon, UK. There was no pre-screening for anti-AAV5 NAb. The toxicity part of the study (main) included 48 animals (24 females and 24 males) in each of the control or AAV5-miHTT dose groups. For each group 16 animals (8 males and 8 females) were necropsied for the 6-month, 3-month, and 1-month timepoints. A satellite group was included for biodistribution and shedding analysis at the 6-month and 1-month time-points; 3 males and 3 females for the control group and 5 males and 5 females for the AAV5-miHTT groups at each time point.

The rats were housed two to four animals per sex to a cage. The animals were housed in a facility that was maintained within temperature and humidity ranges of 20–24 °C and 40%–70%, respectively. The facility was supplied with filtered fresh air and was maintained on an artificial 12 h light 12 h dark cycle. The rats had unrestricted access to food (Teklad 2014C Diet) during the day and to water at any time, except during the night before blood sampling for clinical pathology. The animals were inspected at least twice daily for evidence of ill-health or a reaction to treatment. A detailed weekly physical examination was performed to monitor general health.

### 2.3. AAV5-miHTT Administration

#### 2.3.1. NHP

Prior to surgery, animals were treated with buprenorphine (Buprenovet^®^ 0.02 mg/kg i.m. 0.067 mL/kg). The animals were anaesthetized with intramuscular injections of 5 mg/kg ketamine (Ketamidor^®^ 10%, 0.05 mL/kg) and 0.06 mg/kg medetomidine (Dorbene^®^ 1 mg/mL, 0.06 mL/kg). Indwelling catheters were placed in the vena saphena in both hind legs. The animals were intubated and inhalation anesthesia using 0.8–2 Vol% isoflurane and oxygen was initiated. The Renishaw Drug Delivery System used for intrastriatal implantation of the catheter comprised of an MRI-compatible NHP head fixation frame and fiducial arc (providing 3-dimensional stereotactic references coordinates), implantation trajectory planning software (Neuroinspire™), stereotactic set-up for the head frame, implantation tooling (set of dedicated drills and guides) and a convection-enhanced delivery (CED) catheter set (outer guide, inner guide and catheter).

The frame, with the fiducial arc attached was placed on the heads of the NHPs and the NHPs were moved into a 1.5 T MRI scanner (General Electric, Alliance Medical, Germany). MRI T1 weighted anatomical scan(s) were analyzed using the surgical planning software to determine the trajectory coordinates for catheter implantation as well as the length of the outer- and inner-guides and the catheter. After the MRI, the animals were transported to a surgery suite for catheter implantation where the fiducial arc was removed, the skin lifted from the skull, and the catheter implant site was exposed. The stereotactic set-up in combination with the implantation tooling were used to access the target areas. The guides and catheters were adjusted to the precise length and surgically implanted according to the precise coordinates. Bilaterally, two catheters for CED injection were implanted, one in each caudate nucleus and putamen. The skin was temporarily closed, and the animal returned to the MRI scanner for MRI-guided infusion. Four infusion pumps (B. BraunTM, Melsungen, Germany) were started simultaneously to bilaterally inject the caudate and putamen of each NHP with 100 µL of vehicle alone or with one of three doses of AAV5-miHTT (400 µL total/animal). Infusion rates were gradually increased from 0.8 µL/min to 3 µL/min over 40 min and were maintained at this rate for 30 min. The addition of 1.2 μL ProHanceTM (BIPSO GmbH, Singen, Germany) contrast medium to control vehicle and AAV5-miHTT samples enabled visualization of filling of the target structures by real time MRI scans during administration and at a final dose completion scan. After the procedure, the animals were transferred back to a separate surgery suite to remove the catheters, to close the wounds and recover from the anesthesia.

#### 2.3.2. Rats

Bilateral intra-striatal injections were performed on each rat under anesthesia guided by a stereotaxic apparatus using the coordinates: 0.4 mm rostral to bregma, 3 mm lateral to midline and 4.5 mm from the dura [20] (Supplementary Figure S1). Prior to injection, skin incisions were made on each hemisphere of the skull. Subcutaneous tissue and periosteum were removed, and local anesthesia was applied. A hole was drilled through the skull using a stereotaxic micromanipulator to achieve suitable anterior/posterior and medial/lateral coordinates. A stereotaxic micromanipulator containing a syringe was positioned over the hole and the needle was advanced into the brain according to the coordinates. The animals received 4 µL of control or AAV5-miHTT per hemisphere by slow bolus injection (1 µL/minute) using a kdS 310 Plus, Nanoliter Syringe Pump. Following injection, the needle was left in situ for 2 min before removal. After both injections, the incisions were sutured, and the animals were allowed to recover from anesthesia and returned to the animal unit.

### 2.4. Sampling Schedule and Aassessments

A full list of the clinical assessments performed and their timepoints in NHP and rats are shown in Supplementary Table S1a,b, respectively.

#### 2.4.1. MRI

The MRI scan obtained after completion of NHP dosing was used to determine accuracy of the catheter implantation as well as filling of the target structure. Accuracy of the catheter position was examined by overlaying the dosing MRI with the planned trajectories. The differences in mm were calculated for each implanted catheter. Filling of the target structure was scored blindly by three reviewers based on the ProHanceTM tracer signal. Scoring assessed whether the tracer signal was limited to the striatal structures only, or extended outside the target structures.

MRI assessments to monitor the local response in NHP were performed at 3 and 6 months after dosing. NHP were first anesthetized with ketamine (10 mg/kg) and medetomidine (0.06 mg/kg). MRI was performed using a mobile MR system (1.5 Tesla General Electric, Alliance medical). MRIs were assessed at the German Primate Centre by an NHP brain experienced radiologist. Scoring was performed on T2-weighted images based on a predefined semi-quantitative grading system and scores were reviewed by an independent expert (radiologist). The grading system included the visibility of the injection track, the presence of T2-hyperintensities (areas of longer T2 relaxation times most likely indicating increased local accumulation of fluid), and the shape and size of the T2-hyperintensities. The 3- and 6-month images were compared with the dosing images (T1-weighted images) to locate the injection track and the site of injection.

#### 2.4.2. Necropsy Procedures

Necropsy of rats was performed at 1-, 3- and 6-month timepoints and in NHPs at 6-months (Supplementary Table S1a,b). Macroscopic observations were performed in both studies. All major organs and tissues routinely assessed in toxicity studies were weighed and tissue samples were preserved in appropriate fixatives for sectioning and histopathological examinations or snap frozen for biomolecular analysis. For both species, the brain was subjected to detailed sectioning to obtain specific brain regions. In rats, the brain was divided into distinct brain sections (striatum, cortex, cerebellum) (Supplementary Table S1a). In NHPs, the brain was removed, placed into a brain matrix and was sectioned coronally into 4 mm slices. The brain slices were organized sequentially and identified with a letter designation A to N. For eighty predefined structures, punch samples were taken from 7 brain slices (B, D, F, H, J, L, and N) and snap frozen for biomolecular analysis. The position of the punches was documented on a copy of the brain map corresponding with the slice. The other brain slices (A, C, E, G, I, K, and M) were fixed in 10% neutral buffed formalin (NBF) for sectioning and histopathological examinations.

#### 2.4.3. Histology

Formalin fixed tissues from both NHP and rats were processed to blocks, and slides were hematoxylin and eosin (H&E) stained for microscopic analysis. Immunohistochemistry (Glial fibrillary acidic protein (GFAP) or IBA-1 (Ionized calcium binding adaptor molecule 1)) was used on brain sections only for more detailed examinations. Evaluation was performed by a board-certified toxicological pathologist and peer reviewed.

#### 2.4.4. Quantitative Polymerase Chain Reaction (QPCR) for Vector DNA

Vector DNA concentration levels were measured in cerebrospinal fluid (CSF), body fluids (semen, urine, feces, saliva, nasal secretions plasma and blood) and tissues, including the brain punches and off-target organs from NHP; and in body fluids (urine, feces, saliva, and plasma) and tissues, including brain regions and off target organs from rats. The same validated QPCR method was used for both species and the analyses were performed under GLP.

DNA was isolated from fluids and feces using spin-column methods and from tissues using a magnetic-bead method. The DNA concentration in samples was quantified according to standard operating procedures using a fluorescence-based method. The amount of AAV5-miHTT vector DNA in samples was analyzed by a Taqman Q-PCR using specific primers and probes in duplicate wells and the coefficient of variation of the independent measurements for each sample had to be ≤5%. The inverse log of the mean Ct signal obtained was calculated to obtain copies/reaction using a standard line as calibrator. Subsequently, for shedding samples, the values were corrected for the volume of the sample from which DNA was extracted and for the volume of elution buffer used. In addition, QPCR for an exogenous inhibition control was performed on each sample to detect the presence of QPCR inhibitors. The mean results for fluid samples are reported in copies/mL, for feces in copies/mg and for swabs in copies/swab. For tissue samples, the values were normalized to input DNA per reaction. The mean results for tissue samples are reported in genome copies/μg of DNA.

#### 2.4.5. Reverse-Transcriptase QPCR (RT-QPCR) for miHTT and HTT mRNA

mi*HTT* expression levels were measured in cerebrospinal fluid, body fluids (semen, urine, feces, saliva, nasal secretions plasma and blood) and tissues, including the brain punches and off-target organs from NHP and in body fluids (urine, feces, saliva, and plasma) and tissues, including brain regions and off target organs from rats using a validated RT-qPCR method for both species and the analyses were performed under GLP.

mi*HTT* expression was assessed via a two-step RT-QPCR assay, using a miHTT-24nt reference as a calibration standard, to determine the number of mi*HTT* copies in Cynomolgus monkey and rat RNA. In summary, 10 ng of RNA samples from selected brain punches and off-target tissues by Taqman Q-PCR. As such, the limit of detection (LOD) of the assay was set at 2.5 × 10^3^ copies/µg of RNA.

Additionally, under GLP compliance, a validated one-step, triplex reverse transcription quantitative PCR (RT-QPCR) method was used to assess *HTT* gene expression relative to endogenous reference genes (*GAPDH* and *ATP5B*) in Cynomolgus monkey brain samples. A quality control (QC) sample was used during sample analysis for data normalization.

#### 2.4.6. Luciferase HTT Reporter Assay

AAV5-miHTT was designed to bind to human Huntingtin mRNA without any mismatches; however, it does have one mismatch with NHP *HTT* mRNA. To evaluate the effect of this mismatch on the targeting of mi*HTT*, and therefore lowering of *HTT* mRNA and protein, a luciferase reporter assay was performed. Different luciferase reporter plasmids were made which contained either 1 mismatch, 2 mismatches or no mismatches at all to the miHTT sequence. The 1 mismatch luciferase reporter plasmid with a mismatch at position 10 (C > T) resembles the sequence of the NHP *HTT* mRNA. HEK293T cells were seeded in MW24 plates at 8 × 10^4^ cells/well in 1ml of medium and incubated at 37 °C overnight. The cells were transfected with the different reporter plasmids at 25ng per well and different concentrations of the mi*HTT* construct were transfected for each reporter plasmid. The medium was refreshed after 16 h of incubation. The cells were harvested two days after transfection, and the dual reporter luciferase assay was performed according to manufacturer’s protocol (Promega, E1960). The medium was aspirated and cells washed with 1X PBS, after which the cells were lysed using the Passive Lysis Buffer. The samples were transferred to black 96-wells plates to ensure no leakage of signal to neighboring wells during readout. First the Luciferase Assay Reagent II was added to the wells and luminescence of the firefly luciferase was measured directly, which was ubiquitously expressed and not targeted by the mi*HTT* miRNA. Then the Stop & Glo^®^ Reagent was added to the wells and luminescence of the Renilla luciferase measured directly, which was lowered by the mi*HTT* miRNA. Lowering was assessed by normalizing the Renilla luciferase signal to the firefly luciferase signal of the same well.

#### 2.4.7. Immunoassay for HTT Protein

HTT protein quantification in NHP brain tissue samples was done using an ultrasensitive single molecule counting (SMC) immunossay (Singulex), based on previously described methods [21]. Briefly, pulverized tissue samples were homogenized in ice cold lysis buffer using a FastPrep-96 tissue homogenizer and stored at −80 °C until further testing. The homogenized brain samples were tested in technical triplicates. A combination of 2B7 (Novartis) and MAB2166 (Sigma-Aldrich, St. Louis, MO, USA) were used as capture and detection antibodies, respectively. The protein was quantified with respect to standard curve of a fragment of N-terminal recombinant HTT (human HTT-Q46, 1–548, concentration range 15.6 fM to 3800 fM). All analyses were performed at IRBM (Pomezia, Italy).

## 3. Results

To determine the safety of intrastriatal administration and biodistribution of AAV5-miHTT in NHP, Cynomolgus monkeys were randomized to a single (one-time) bilateral intrastriatal administration of vehicle control or AAV5-miHTT at 2 × 10^12^, 7 × 10^12^ or 2 × 10^13^ genome copies (gc)/animal. Each dose group included 3 males and 3 females, resulting in a total of 24 animals in the study. Every animal was injected bilaterally in the caudate nucleus and putamen under MRI guidance by convection enhanced delivery (CED) and animals were followed for 6 months. The administration procedure in NHP was performed using a protocol and materials that are directly translatable to the clinical situation. A study in rats was also performed to have multiple interim timepoints to evaluate biodistribution and safety. Sprague-Dawley rats were treated with either vehicle control or AAV5-miHTT at 4 × 10^9^, 4 × 10^10^, 4 × 10^11^ gc/animal by stereotactic injection into the striatum. The rats were followed for 1-, 3- or 6-months after injection. Biodistribution of the vector and the miHTT transgene was assessed for both species, a full list of toxicology parameters was evaluated, and histopathology was performed on all major organs, including the brain as target organ.

### 3.1. Accurate Catheter Implantation and Good Target Structure Coverage in NHP

**Figure 1:**
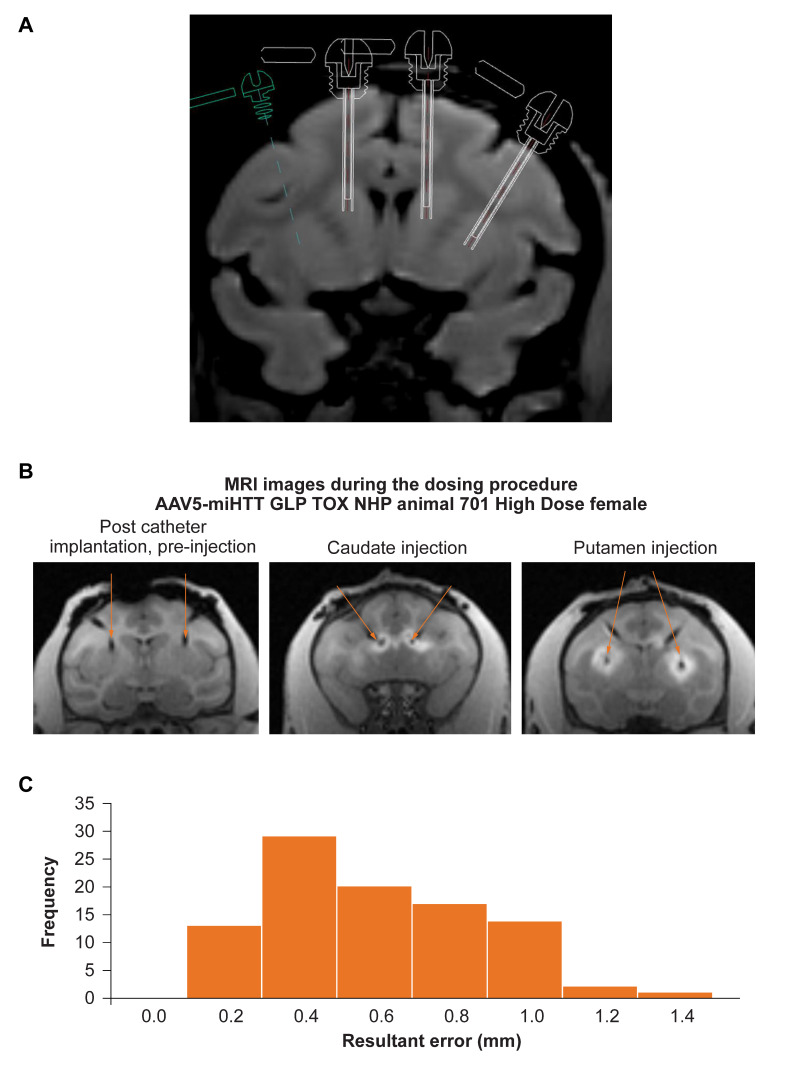
Successful MRI guided convection enhanced delivery of AAV5-miHTT to non-human primates (NHP). (**A**) The trajectory of the cannulas going into the target brain regions was planned based on individual animal MRI to calculate the exact coordinates of the trajectory. (**B**) Intra-operative MRI allowed close monitoring of the injections, with the left panel showing catheter placement before the injection (indicated by the orange arrows), and the middle and right panel showing the gadolinium signal of caudate nucleus and putamen being injected, respectively. (**C**) The error between the planned and actual injection location of all injections was measured based on the gadolinium signal. Nearly all (97%) injections were within 1 mm of the planned location within the target brain area, showing high accuracy. The graph shows how frequently (out of 96 in total) the resultant error of a certain distance was recorded.

A total of ninety-six catheters were implanted in 24 NHP by MRI guided CED administration of AAV5-miHTT using specialized planning software (Figure 1a). All catheters were successfully implanted into the intended target regions (head of the caudate nucleus and the rostral part of putamen of both hemispheres) (Figure 1b) with an average standard error mean of 0.5 mm ± 0.03 (which is the size of one voxel in the images) and nearly all (97%) catheters were implanted within 1 mm of the planned target, which is well within the accepted range (Figure 1c). The tracer signal to confirm correct placement and injection was present in the target areas in 97% of the injections. The tracer signal was primarily found in the target areas and in most NHPs, distribution of tracer signal was also recorded in other peri-striatal structures (internal capsule, globus pallidus) as well as outside the striatal structures into adjacent structures (e.g., external capsule, corpus callosum and in a few cases into cortical structures). Only in one female NHP in the AAV5-miHTT 2 × 10^12^ gc group almost no tracer signal was detectable in the left caudate (1 out of 96 injections). The dose completion MRI confirmed accurate placement of the catheters, resulting in an excellent filling of target structures.

### 3.2. Structural MRI in NHP to Assess Safety of Administration Procedure

**Figure 2:**
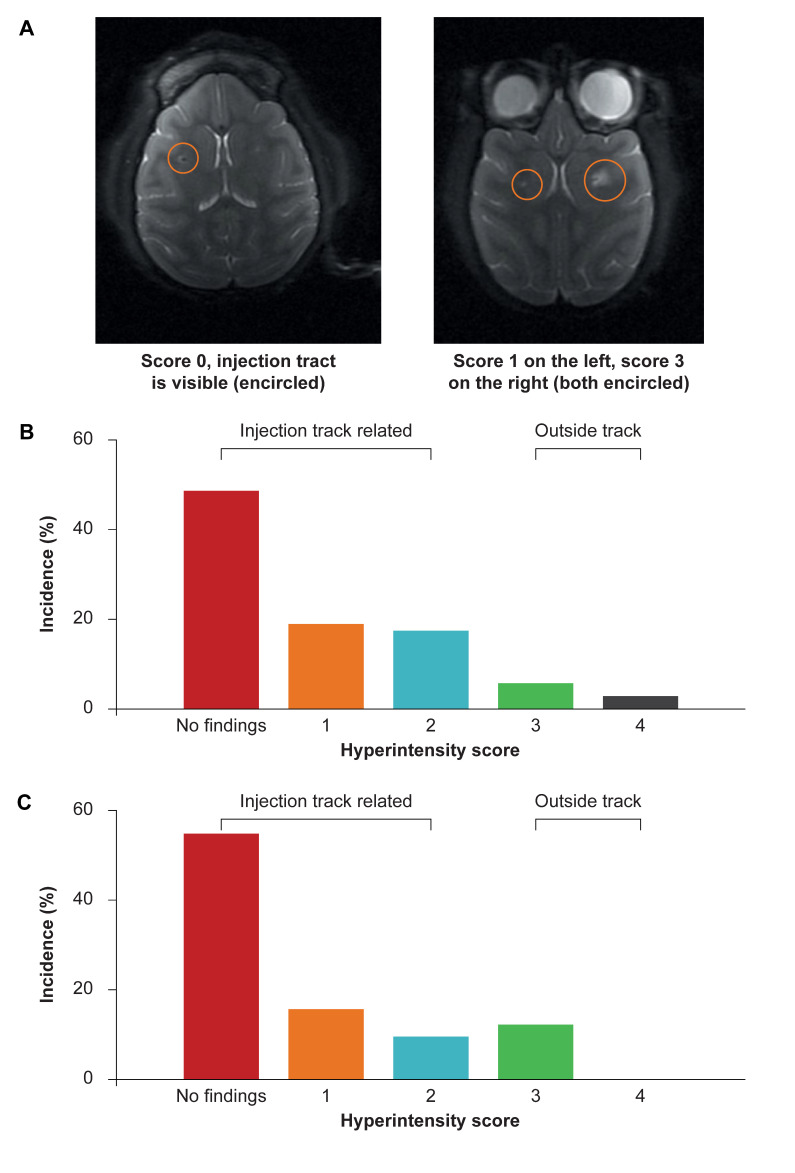
T2 hyperintensities. Seen on MRI, these are an indication of altered fluid content (e.g., blood, edema, inflammation) compared to the surrounding area. Brain MRI from NHP taken 3 and 6 months after dosing indicated that for most injections no hyperintensity was observed and for the injection tracts where hyperintensity was observed in the MR image, a tendency to recovery over time was seen in NHP. (**A**) Examples of hyperintensities seen on MR images. Score 0: track visible, no T2-hyperintensities. Score 1 and 2: T2-hyperintensities directly related to the injection track. Score 1: very small hyperintense dot near the end of the track or incomplete T2-hyperintense circle around the track. Score 2: a small hyperintense dot near the end of the track or complete T2-hyperintense circle around the track. Score 3 and 4: T2-hyperintensities extending outside the area of the injection track. Score 3: within injected area. Score 4 = extending outside the injected area as compared with the dosing MRI. (**B**) Hyperintensity scores 3-months after dosing. (**C**) Hyperintensity scores 6-months after dosing, (incidence in % cases).

MRIs were recorded at 3- and 6-months post-injection to evaluate the local response at the injection sites. Structural MRIs showed no abnormalities at 3 months in 49/96 injections (51%) and in 56/96 injections (58%) at the end of the study (Figure 2). Some of the injections showed T2-hyperintensities (bright areas in T2 weighted images) that were directly associated with the injection track or target area in 40% (n = 38) at 3 months and in 28% (n = 27) at the end of the study. T2-hyperintensities extended beyond the very narrow region surrounding the area of injection at 3 months in 9/96 (9%) and were observable at study end in 13/96 injections (14%). These T2-hyperintensities were observed in all groups, including control animals treated with vehicle only. There was no correlation between the dose and the incidence or size of the T2-hyperintensity. In addition, the T2-hyperintensity diminished over the study period and at study end was either less or similar as compared to the interim evaluation. These structural MRI assessments suggest that the initial tissue disruption caused by the neurosurgical installation of the catheter recovers over time. There did not appear to be a correlation between the intraparenchymal injected dose of AAV5-miHTT and T2 dose-dependent hyperintensities observed using MRI.

### 3.3. Widespread Vector DNA and miHTT Transgene Distribution in Brain Areas Associated with HD Pathology

**Figure 3:**
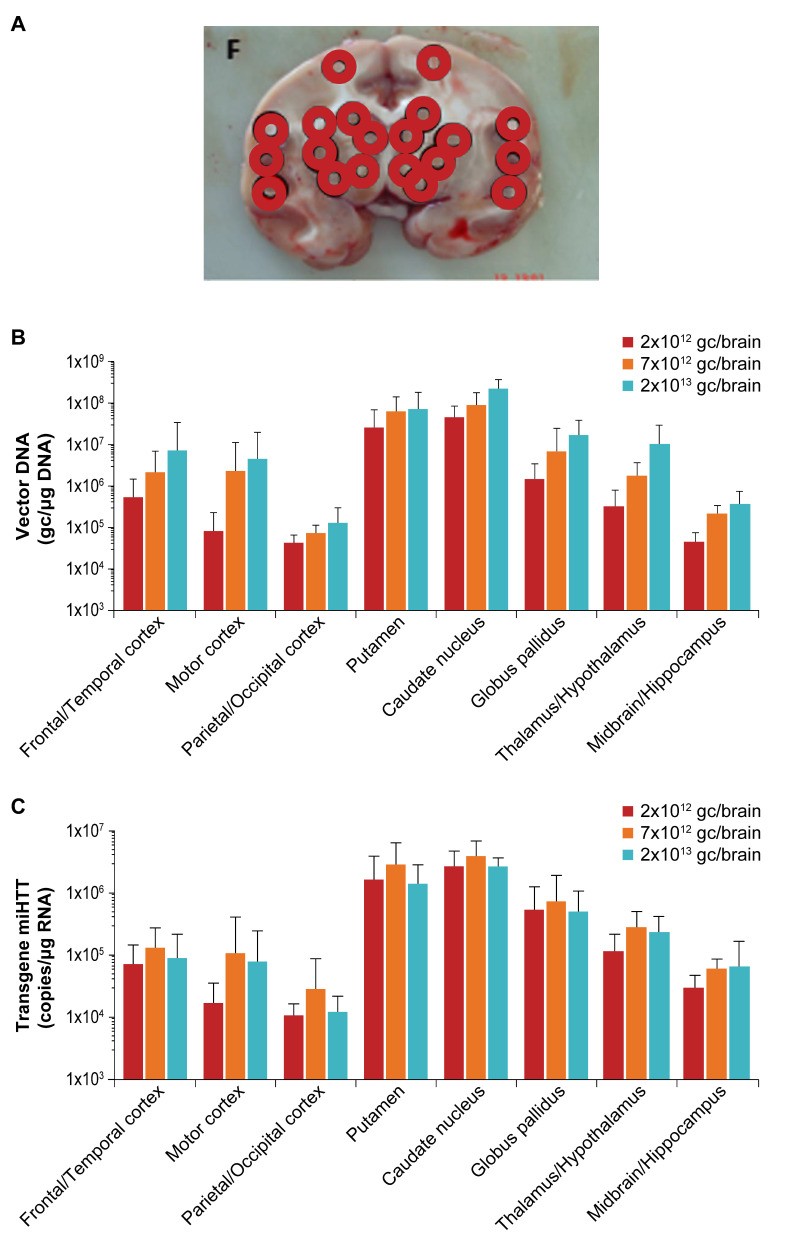

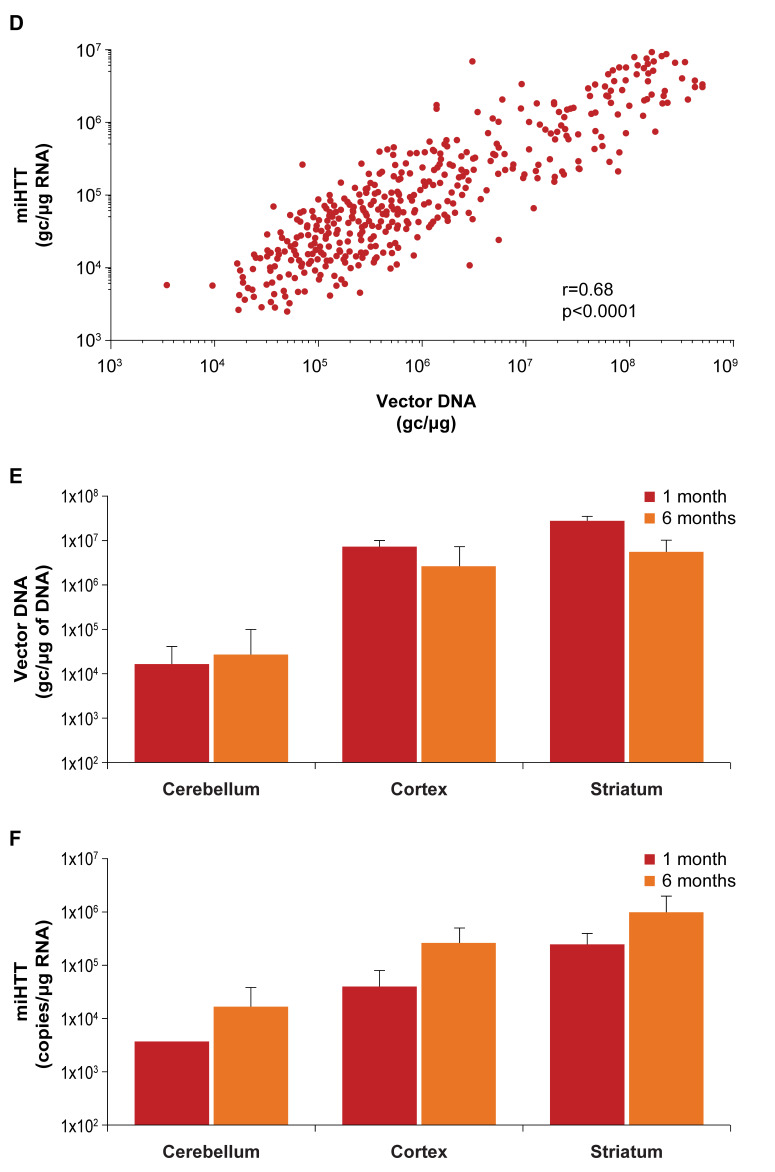
Brain punch analyses. Punches were collected from several brain slices 6 months after injection with AAV5-miHTT and each punch was divided into 3 aliquots to allow different analyses for NHP. For the rats, different brain sections were used for vector DNA and mi*HTT* analysis. (**A**) An example of a brain map showing where the different punches were taken from brain slice F. (**B**) Vector DNA levels of the brain punches shown as an average (with standard deviation (SD)) of genome copies found per brain region and dose group. (**C**) mi*HTT* transgene expression of the brain punches shown as an average (with SD) of mi*HTT* copies per brain region and dose group. (**D**) Correlation between vector DNA and mi*HTT* transgene expression in NHPs. (**E**) Vector DNA levels in different brain regions of rats shown per dose group and per timepoint as average (with SD). (**F**) mi*HTT* expression in different brain regions of rats shown per dose group and per timepoint as average (with SD). Only one cerebellum sample was above the lower limit of quantification (LLOQ) for mi*HTT* (2.5 × 10^3^ copies/µg of RNA) at 1 month.

Dose-related transduction of the target structures was observed in NHP at 6 months following AAV5-miHTT administration. Brain samples throughout the entire brain were taken and the level of vector DNA was determined (Figure 3a). The highest levels of vector DNA were observed in the highest dose group at the site of injection in the putamen and caudate nucleus of NHP reaching up to 7.3 × 10^7^ gc/µg gDNA and 2.2 × 10^8^ gc/µg gDNA, respectively (Figure 3b). Vector DNA was also observed in other areas of the brain including the cortex, thalamus, and hippocampus (Figure 3b). This indicates that even though the AAV5-miHTT was only injected into the striatum, it spreads throughout the entire brain. The expression of mi*HTT* was also assessed in each brain region, with levels up to 2.9 × 10^6^ copies/µg RNA in putamen and 3.8 × 10^6^ copies/µg RNA in caudate nucleus (Figure 3c). Even though mi*HTT* expression did not show a strong dose-dependent effect in the high dose, there was a strong correlation between brain vector DNA and mi*HTT* transgene expression (r = 0.68, *p* < 0.0001) (Figure 3d). In the rat model, vector DNA expression was highest in the striatum up to 3.4 × 10^7^ gc/µg gDNA (putamen and caudate nucleus combined) followed by the cortex (up to 9.3 × 10^6^ gc/µg gDNA) and was lower in the cerebellum (5.1 × 10^4^ gc/µg gDNA) (Figure 3e). mi*HTT* expression followed the same pattern with highest levels in the striatum (up to 1.2 × 10^6^ copies/µg RNA) (Figure 3f). Notably, vector DNA levels were highest at 4 weeks post-injection and slightly lower levels were found 6 months after injection, while mi*HTT* levels were highest at the 6-month time point. The same brain punches as were used for the DNA and miRNA analysis were used for *HTT* mRNA and protein analysis in NHP, to determine the level of *HTT* protein in different brain structures and to compare to control animals. An in vitro luciferase reporter assay showed that 1 nucleotide mismatch between the mi*HTT* and NHP *HTT* mRNA at position 10 of the microRNA binding site abrogated the binding of mi*HTT* and thus resulted in no measurable lowering of monkey HTT (Supplementary Figure S2). Results from all dose groups in the NHP study showed no lowering of *HTT* mRNA or HTT protein after AAV5-miHTT treatment, when compared to the vehicle treated controls in any of the brain regions (Supplementary Figure S3). This confirms the high specificity of mi*HTT* and thereby strongly limits the potential off-target effects of the microRNA [22]. Analysis of both NHP and rat brain regions showed high levels of vector DNA and mi*HTT* expression that were sustained over 6 months and were widespread throughout the brain, thereby confirming high transduction of brain regions relevant for HD and subsequent expression of the transgene in those regions.

**Figure 4:**
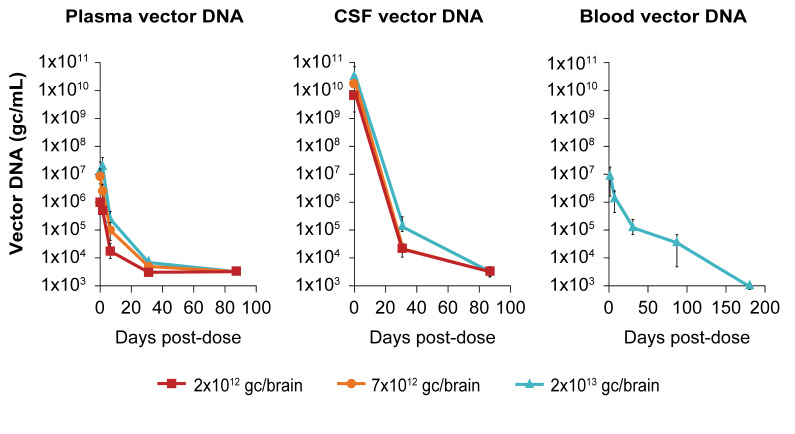
Shedding of vector DNA in body fluids in NHP after AAV5-miHTT injection. Vector DNA was found at high levels within 1 h after dosing in blood, plasma and cerebrospinal fluid (CSF), which declined over time until reaching levels close to the LLOQ (25 copies/reaction) by 3 (plasma and CSF) or 6 months (blood) after dosing. In feces, urine and semen vector DNA was found at day 7 and 1 months after dosing, but no vector was present after 3 months (data not shown). Vector DNA was present in saliva and nasal swabs at 3 months post dose, but not at 6 months (data not shown).

Even though administration of the AAV vector is directly into the brain tissue, we anticipated that some of the vector DNA would not remain within the brain parenchyma. Different body fluids were collected following dosing to investigate this. The levels of vector DNA in the CSF in all AAV5-miHTT-treated NHP were approximately 1 × 10^10^ gc/mL at 1-h post injection. At the same timepoint, vector DNA was also found in blood and plasma, but at a lower level (up to 1 × 10^7^gc/mL) (Figure 4). One week after dosing, vector DNA was found in blood, plasma, feces, urine, nasal swabs and saliva. These levels declined at 1 month after dosing and by 3 months after dosing the vector DNA levels were below the limit of detection for plasma, feces, urine and semen. Six months after dosing, vector DNA was no longer detectable in any of the body fluids. In the rat model vector DNA was found at high levels in plasma only 4 h after dosing. Vector DNA was also detected in urine and saliva at day 3, but for all body fluids the level of vector DNA decreased over time until reaching close to the limit of quantification by 3 months after dosing (Supplementary Figure S4). These data show that after intrastriatal delivery of AAV5-miHTT the vector is shed into CSF and blood very rapidly, which indicated that initially the vector was able to spread systemically and was not limited to the CNS. However, this was not sustained and by 3 months after dosing vector DNA was near or below detection levels in all body fluids. For both rats and NHP the level of miHTT in off-target tissue was always at least 100 times lower than the levels found in the target brain region (data not shown).

### 3.4. No Impact on General Safety Measures Following AAV5-miHTT Treatment in NHP or Rats

During the in-life phase of the studies, many different safety endpoints were recorded. There were no observable changes in clinical signs, neurological assessment, growth, cardiovascular endpoints, body temperature, ophthalmoscopy, hematology or clinical chemistry associated with AAV5-miHTT treatment in NHPs (Supplementary Table S3). Similarly, there were no observable changes in clinical signs, body weight, food and water consumption, body temperature, hematology, blood chemistry, or post-mortem organ weights associated with AAV5-miHTT treatment in rats. A modest and transient increase in plasma IL-6 was observed on Day 2 after dosing in the NHP control and treatment groups. No other cytokine related findings were observed in either NHP or rats. As would be expected, NAb to AAV5 were present in the blood plasma of both NHP and rats treated with AAV5-miHTT 6 months after dosing (data not shown). For rats both at 1 and 6 months after dosing and in NHP at 6 months after dosing macro and microscopic evaluations were performed on the major organs. No treatment related histopathology findings were observed in non-target tissues in either species. The general safety measures performed during the in-life phase and after necropsy showed no indication of AAV5-miHTT treatment related toxicity in the rats or NHP up to 6 months after dosing.

### 3.5. No Evidence of AAV5-miHTT Related Neuropathology

Histopathology of the brain was performed in both studies to investigate the effect of the injection needle itself and the effect of AAV5-miHTT delivery to the brain. In NHP, a granulomatous inflammatory response was found at all injection sites in all groups and was observed to a similar extent in AAV5-miHTT and vehicle treated control animals. Slight perivascular cuffing or satellitosis was also observed in all NHP regardless of treatment. In rats, intrastriatal bilateral injection of either vehicle control or AAV5-miHTT was associated with inflammatory changes/hemosiderosis/gliosis and a low level of vacuolation of nervous tissue. Changes were observed in control and treated animals of both sexes and progressively diminished by 6-months post-administration. There was an increased number of astrocytes (positive cells for GFAP) and microglial cells (positive cells for IBA-1) associated with the needle tract in both control and AAV5-miHTT treated rats (Supplementary Table S4). The observed changes around the needle tract in both NHP and rat studies were related to the needle placement procedure rather than AAV5-miHTT since they were also observed to a similar extent in the vehicle treated animals. Taken together with the results from the structural MRI, in-life safety parameters and histopathology, these data demonstrate the excellent safety profile and tolerability of AAV5-miHTT treatment in rats and NHP.

## 4. Discussion

Having demonstrated proof of concept for AAV5-miHTT in both small and large animal models [12,14,16,17], the objectives of these GLP studies were to examine the safety, tolerability, biodistribution, mi*HTT* expression and vector DNA shedding of intrastriatal administration of AAV5-miHTT in NHP and SD rats to build on previous preclinical data. These studies provide valuable information on the dosing procedure, dose effects, tolerability, target and non-target distribution and vector shedding to enable the translation of AAV5-miHTT into clinical studies.

A key and novel finding from this study is that real-time, MRI-guided, CED of a high concentration of AAV5-miHTT to the putamen and caudate nucleus was well tolerated and reproducible in NHP. Ninety-six catheters were successfully implanted in 24 animals without adverse surgical events. AAV5-miHTT administration hit the target area with excellent coverage of the target structure shown by real-time tracer signal and post-mortem biodistribution analysis of vector DNA. All in-life clinical observations, including neurological examinations in NHP, and analyses were unremarkable in both animal models suggesting that the treatment was well-tolerated with an acceptable safety profile. Another innovative aspect of this study was the use of MRI in NHP 3- and 6-months following AAV5-miHTT administration to examine healing of the brain around the injection track as well as T2 hyperintensities related to the procedure. These data indicated cannula track recovery and a reduction in areas of T2 hyperintensity during the follow up period in NHP. As treatments such as AAV5-miHTT enter the clinic, MRI should provide a useful method to determine surgical recovery following intrastriatal administration. Histopathology in both models revealed only local procedure-related findings. These were shown to be transient as rats were evaluated at 1-, 3- and 6-months after dosing, and showed recovery. Taken together, these safety evaluations indicate that the intrastriatal delivery of AAV5-miHTT to the striatum is well tolerated in both rats and NHP.

Different AAV vectors have been utilized to target the CNS in NHP and in human patients with intraparenchymal delivery. A study comparing transduction efficacy of AAV1- 2-, 3-, 4-, 5- and 6-GFP infused into the substantia nigra and caudate nucleus of NHP concluded that AAV5 was the most efficient vector, as it transduced significantly more cells than the other serotypes, and was effective at transducing both neurons and glial cells [23]. This study also investigated tolerability of the administration procedure and all of the NHP survived the study period (30 days), were in good health and had no evidence of behavioral abnormalities, loss of appetite, weight loss, or other adverse signs following surgery [23]. More recently, another group used an MRI-guided CED approach to infuse AAV1- or AAV2-GFP into the putamen and caudate nucleus of NHP [24]. They demonstrated accurate catheter placement, target coverage and expression of GFP in the putamen and caudate nucleus and other brain areas. Furthermore, studies involving multiple, intracerebral injections of AAV vectors have been completed in humans. This includes a Phase I clinical trial in subjects with Canavan disease [25,26], and a Phase I clinical trial in subjects with ceroid lipofuscinosis type 2 [27,28,29]. Phase I gene-therapy trials involving one-time injections of AAV vectors directly into the brain have also been performed in adult subjects with Parkinson disease [30,31,32,33]. In a Sanfilippo B Phase I/II trial of rAAV5-NaGlu (AMT110-CD-001 [34]), the vector was administered directly into the cortex through eight different injection sites; two in the posterior fossa targeting the white matter of the cerebellar hemispheres and six supratentorially targeting the white matter adjacent to the putamen. No signs of inflammation, edema, or necrosis were detected up to 30 months after treatment [34]. Overall, there were no significant adverse events due to the AAV gene-therapy treatment approach in any of these trials. These studies support the use of AAV5 for CNS indications and complement our findings of good tolerability of intraparenchymal delivery of AAV.

The widespread distribution of vector DNA throughout different brain regions in both the NHP and rat models demonstrate the ability of intra-striatally administered AAV5-miHTT to highly transduce the putamen and caudate nucleus (striatum), key neuroanatomical areas involved in the pathology of HD, as well as spread to other areas of the brain such as the cortex, which are further from the injection site. There was a strong linear correlation between vector DNA and mi*HTT* expression in NHP brain tissue, showing widespread expression of the microRNA targeting the Huntingtin mRNA. mi*HTT* persistence in the brain was also demonstrated in rats, as expression was numerically higher in the striatum, cortex and cerebellum at 6 months versus 1 month. Our data shows that the highest levels of vector DNA and mi*HTT* were found in the striatum in both animal models, with spread to other brain regions. This is in contrast to alternative approaches, such as the intrathecal administration of ASO, which appear to result in greater distribution and therapeutic effect in the cortex when compared to the striatum [11,13].

The biodistribution and mi*HTT* expression data from this study compliments findings from preclinical small and large animal models of HD. The levels of micro RNA targeting Huntingtin mRNA in the current studies was comparable to the previously reported studies which showed that AAV5-miHTT administration leads to suppression of mutant HTT protein production, reduction of mutant HTT aggregation and functional improvements in motor coordination [14,16,17]. As expected, due to a single nucleotide difference in *HTT* mRNA between NHP and humans, AAV5-miHTT had no impact on *HTT* mRNA or HTT protein expression in the brains of NHP (Supplementary Figure S4) in the current study. This supports the specificity and lack of off-target activity of AAV5-miHTT reported elsewhere [22].

## 5. Conclusions

In conclusion, bilateral intrastriatal CED administration of AAV5-miHTT was reproducible and well tolerated in both NHP and rats with the highest dose being the No Observable Adverse Effect Level (NOAEL). For NHP, real-time MRI was used as a tool to guide catheter placement, dosing accuracy and post-treatment follow up. Post-treatment MRIs in NHP demonstrated ongoing healing of the injection track and surrounding brain tissue. Histopathological findings in both rats and NHP appeared to be associated with the intrastriatal infusion procedure itself rather than AAV5-miHTT treatment, and recovery of these findings was seen over time. AAV5-miHTT achieved persistent high-level expression of vector DNA and mi*HTT* transgene. Given that NHP are considered to provide a good animal model for humans, the excellent vector delivery, tolerability of the procedure, high vector levels at target, widespread distribution of vector to other brain areas and positive safety profile support the evaluation of AAV5-miHTT in a human clinical study. Based on these GLP safety and biodistribution studies and the previously presented efficacy data in HD models, a phase 1/2 clinical study of intrastriatal administration of AAV5-miHTT in individuals with HD has recently commenced (NCT04120493).

## Data Availability

The data presented in this study are available on request from the corresponding author.

## Supplementary Materials

The following are available online at https://www.mdpi.com/2076-3425/11/2/129/s1, Figure S1: Injection site locations in rats; Figure S2: The luciferase reporter assay showed a clear dose-dependent lowering of the luciferase reporter that has no mismatch to the AAV5-miHTT target sequence; Figure S3: (a) HTT mRNA gene expression and (b) HTT protein levels in the brain of NHP; Figure S4: Rat shedding; Table S1: Clinical measures in (a) NHP study and (b) rat study; Table S2: mi*HTT* expression in off-target organs (a) NHP and (b) rat; Table S3: Clinical observations following AAV5-miHTT treatment in NHP or rats; Table S4: Microscopic examination of the brain showing astrocytes and microglial cell increases associated with the needle tract in rats 1 month after injection with vehicle or AAV5-miHTT.
