# Supplementary material for: Intrastriatal Administration of AAV5-miHTT in Non-Human Primates and Rats Is Well Tolerated and Results in miHTT Transgene Expression in Key Areas of Huntington Disease Pathology
Source: Brain Sci. 2021 Jan 20;11(2):129. doi: 10.3390/brainsci11020129 (PMC7908995; doi:10.3390/brainsci11020129)
Supplement: Supplementary file 1 [file brainsci-11-00129-s001.zip › brainsci-1035997-Sup/Spronck Brain Sciences Fig S2_JAN 10.pdf]

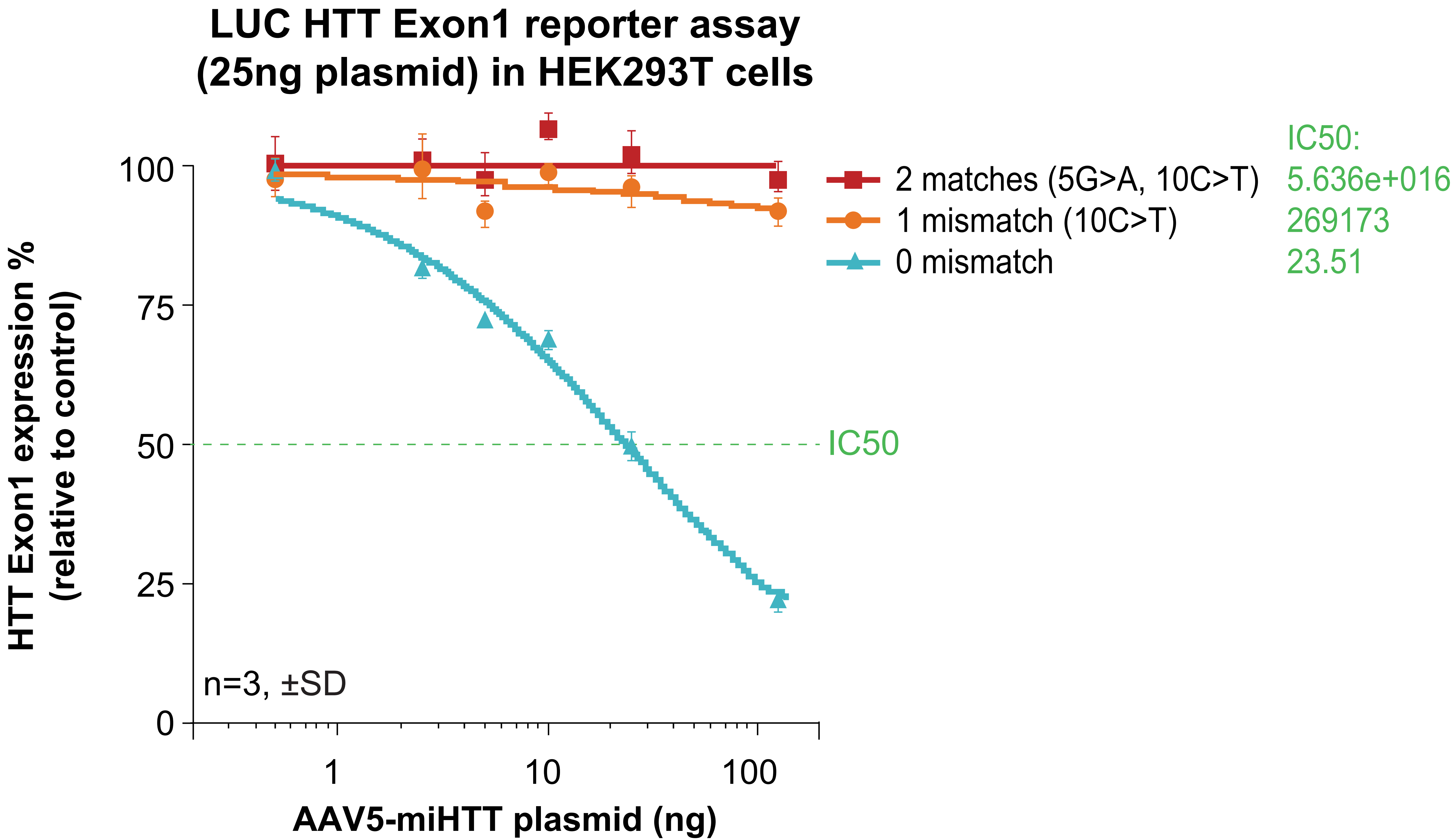

Supplementary figure 1. The Luciferase reporter assay showed a clear dose-dependent lowering of the luciferase reporter that has no mismatch to the AAV5-miHTT target sequence. The reporter with one mismatch, as is the case in the NHPs, did not show any lowering. Same holds true for the control reporter construct with 2 mismatches, confirming the validity of this assay.
