# Supplementary material for: Intrastriatal Administration of AAV5-miHTT in Non-Human Primates and Rats Is Well Tolerated and Results in miHTT Transgene Expression in Key Areas of Huntington Disease Pathology
Source: Brain Sci. 2021 Jan 20;11(2):129. doi: 10.3390/brainsci11020129 (PMC7908995; doi:10.3390/brainsci11020129)
Supplement: Supplementary file 1 [file brainsci-11-00129-s001.zip › brainsci-1035997-Sup/Supplementary methods.pdf]

Supplementary methods

NHP

Cytokine analysis

Blood samples were tested for interferon-gamma (IFN-γ), interleukin-4 (IL-4), tumor-necrosis factor alpha (TNF-α), IL-6, monocyte chemoattractant protein 1 (MCP-1), IL-8, IL-1β, IL-10, IL-2. The analysis was performed using a MilliPlex MAP kit (Merck Millipore, Darmstadt, Germany), according to the manufacturer's instructions. The test has been validated for use with cynomolgus monkey serum. Analyses were conducted according to the current version of the Analytical Procedure AP 16- 011 (EMD Millipore Milliplex Map Kit). Samples were analyzed in 3 approved analytical batches.

Clinical chemistry

Serum samples derived from 1.5 mL of whole blood from fasted animals (except on Day 2) were tested for the parameters listed in Supplementary table 1a.

Cardiovascular investigations

An eight-lead ECG measurement (Leads I, II, III, aVR, aVL, aVF, V1, and V2) was performed and the following parameters were measured. Time Measurements (V2): Heart rate (b/min), RR, PR, QRS, QT, corrected QT (QTcB) interval (msec) From chest lead V2, an average of at least five beats (consecutive if possible) was analyzed. Systolic, diastolic, and mean arterial pressures (mmHg) were recorded in all animals by high definition oscillometry (HDO) method.

Neurological examinations

Physical and neurologic examinations were performed on all unsedated animals twice during the predose phase, daily during the first week after dosing (from Day 2 onwards), and every 2 weeks thereafter, and prior necropsy to determine any adverse effect on central nervous system function.

Physical examinations included abdominal palpation, body temperature, and heart (functional) and lung (functional) auscultation. Neurologic examinations included general sensorimotor aspects, cerebral reflexes (pupillary and orbicularis oculi), and spinal reflexes (patellar and anal).

Hematology

The hematology parameters listed in Supplementary table 1a were analyzed in 0.5 mL blood samples taken into tubes with EDTA anticoagulant. Prothrombin time and activated partial thromboplastin time were analyzed in blood collected into citrate anticoagulant.

Urinalysis

Urine samples (after approximately 2 hours without food) were collected from all animals once during the predose phase, during Weeks 4 or 5, 12 or 13 of the dosing phase, and in Weeks 25 or 26. The following parameters were analyzed: volume, ketones*, specific gravity, urobilinogen*, pH, bilirubin*, leukocytes*, occult blood*, nitrite*, color, protein*, clarity, and glucose*. For parameters highlighted by an asterisk, a qualitative determination was made with concentrations transferred in a grading system without units.

Rat

Cytokine analysis

Rat K_2EDTA plasma samples were analysed using the Bio-Rad Bio-Plex 200 reader for the determination of the concentrations of cytokines listed in Supplementary table 1b.

Clinical chemistry

Animals were held under light general anesthesia induced by isoflurane. Blood samples (nominally 0.7 mL) were withdrawn from the sublingual vein and collected into tubes containing lithium heparin as anticoagulant. After separation, the plasma was examined using a Roche P Modular Analyzer for the parameters listed in Supplementary table 1b

Hematology

Animals were held under light general anesthesia induced by isoflurane. Blood samples (nominally 0.5 mL) were withdrawn from the sublingual vein, collected into tubes containing EDTA anticoagulant and examined for the parameters listed in Supplementary table 1b using a Bayer Advia 120. Additional blood samples (nominally 0.5 mL) were taken into tubes containing citrate anticoagulant and examined using a Stago STA Compact Max analyzer for prothrombin time and activated partial thromboplastin time.
