# Supplementary material for: Intrastriatal Administration of AAV5-miHTT in Non-Human Primates and Rats Is Well Tolerated and Results in miHTT Transgene Expression in Key Areas of Huntington Disease Pathology
Source: Brain Sci. 2021 Jan 20;11(2):129. doi: 10.3390/brainsci11020129 (PMC7908995; doi:10.3390/brainsci11020129)
Supplement: Supplementary file 1 [file brainsci-11-00129-s001.zip › brainsci-1035997-Sup/Table S1.pdf]

Supplementary table 1. Clinical measures in a) NHP study and b) rat study

a) Clinical measures and timepoints in NHP study

Measures	Frequency
**Living animals**	
Vector DNA in CSF	Day 0, 1, month 1, 3, 6
Vector DNA in bodily fluids (semen, urine, feces, saliva, nasal swab, blood)	CSF: pre-dose, day 1 and 1-, 3-, 4- and 6-months after dosing<br>Semen: 1-, 3- and 6-months after dosing<br>Urine, feces: day 7 and 1-, 2-, 3- and 6-months after dosing<br>saliva, nasal swab: day 7 and 1-, 2- and 3-months after dosing<br>Blood and plasma: pre-dose, day 1, day 2, day 7 and 1-, 2-, 3- and 6-months after dosing
Morbidity and mortality/clinical examination	Twice daily
Post-dose observations	Once on Day 1, and during cage checks (morning and afternoon)
Body weight	Twice pre-dose, weekly during dosing and the day prior to termination
Physical and neurological examinations	Twice pre-dose, daily during the first week postdosing (from Day 2 onwards), every 2 weeks thereafter, and prior to termination.
Ophthalmic examination	Once during the pre-dose phase, 1, 3 and 6 Months after dosing
Cardiovascular investigations (electrocardiography, blood pressure)	Once during the pre-dose phase, 1, 3 and 6 Months after dosing
Respiratory rate	Once during the pre-dose phase (morning), Day 1 (prior surgery/dosing), day 2, day 3 and 1-, 3- and 6-Months after dosing
Magnetic Resonance Imaging (MRI) Assessment	3 and 6 Months after dosing
Hematology (Hct, Hb, RBC, Retic, MCH, MCHC, MCV, WBC, N, L, E, B, M, LUC, Plt)	Twice during the pre-dose phase, Day 2 (non-fasted), Day 8 and 1-, 3- and 6-Months after dosing
Coagulation (Prothrombin Time [PT], Activated Partial Thromboplastin Time [APTT])	Twice during the pre-dose phase, Day 2 (non-fasted), Day 8 and 1-, 3- and 6-Months after dosing
Clinical chemistry (ALP, ALT, AST, Bili, Urea, Creat, Gluc, Chol, Trig, Na, K, Cl, Ca, Phos, Total Prot, Alb, A/G Ratio, GLDH, GGT, Glob	Twice during the pre-dose phase, Day 2 (non-fasted), Day 8 and 1-, 3- and 6-Months after dosing
Urinalysis	Once during the pre-dose phase, Months 1, 3 and 6
Cytokine analysis (IFN-γ, IL-4, TNF-α, IL-6, MCP-1, IL-8, IL-1β, IL-10, IL-2)	Once pre-dose (week before first dose), Days 2, 4, 8
**Necropsy**	
Macroscopic examination	Post-termination
Organ weights and tissue sections	Post-termination
AAV5 nAb	Post-termination
Vector DNA, mi*HTT* and HTT protein in tissues (Adrenals, Brain (punches from all brain regions), deep cervical lymph nodes, eyes, bone marrow femur, mammary glands, heart, ileum, kidney, liver, lungs, ovaries, pancreas, pituitary, prostate, salivary glands, seminal vesicles, skeletal muscle, spinal cord, spleen, testes, thymus, thyroid, uturus/cervix)	Post-termination Tissue collected (male/female where applicable)

B) Clinical measures and timepoints in Rat study

Measures	Frequency
**Living animals**	
Clinical observation (visual check for evidence of ill health or treatment reaction)	Twice daily

Detailed observations around dosing	Pre-dose (surgical site observation), 2 and 4 hours after dosing and as late as possible in the working day following dose administration
Surgical site assessment for site reactions or healing	Daily until any reaction was resolved or the surgical site was healed, and at termination
Body Weight	Weekly starting the week before treatment
Food Consumption	Weekly starting the week before treatment
Water consumption	The week before treatment Weeks 1, Months 1,2, 3 and 6
Body temperature	Pre dose, 24 h post dose, Day 4, Months 1, 3 and 6
Hematology (Hct,* Hb, RBC, Retic, MCH,* MCHC,* MCV, RDW, WBC, N, L, E, B, M, LUC, Plt)	Day 8, Months 1, 3 and 6
Blood chemistry (ALP, ALT, AST, Bili, Urea, Create, Gluc, Chol, Trig, Na, K, Cl, Ca, Phos, Total Prot, Alb, A/G Ratio)	Day 8, Months 1, 3 and 6
Vector DNA in body fluids (urine, feces, saliva, plasma)	Day 1 (4- and 24-hours post dose), Day 8, Months 1, 3 and 6
Assessment of Anti-AAV5 Antibody Level	Prior to termination
Cytokine levels	Day 1 (4- and 24-hours post dose), Days 4, 8 and 15
**Necropsy**	
Macroscopic examination	Post-termination tissues
Organ weights and tissue sections	Post-termination tissues
Bone marrow smear	Post-termination tissues
Vector shedding in CSF and Vas Deferens	Post-termination
Vector DNA and mi*HTT* in tissues<br>(Adrenals, bone with marrow, brain (striatum, cortex, cerebellum, rest), cecum, colon, duodenum, epididymides, eyes, harderian glands, heart, ileum, jejunum, kidney, liver, lung, axillary and deep cervical lymph nodes, ovaries, pancreas, prostate, seminal vesicle, skeletal muscle, skin with mammary glands, spinal cord, spleen, stomach, testes, thymus, bladder, uterus/cervix, vagina)	Post-termination tissues:

* Derived values calculated in ClinAxys (Clinical Systems Ltd., Cambridgeshire, UK).
Abbreviations:
A/G ratio; albumin/globulin ratio; Alb, albumin; ALP, alkaline phosphatase; ALT, alanine aminotransferase; AST aspartate aminotransferase; B, basophils; Bili, total bilirubin; Ca, calcium; Chol, total cholesterol; Cl, chloride; Creat, creatinine; CSF, cerebrospinal fluid; e, eosinophils; GGT, gamma glutamyl transferase; GLDH, glutamate dehydrogenase; GLOB, globulin; Gluc, glucose; Hb, hemoglobin concentration; Hct,*hematocrit; IFN-γ, Interferon-gamma; IL-1β, Interleukin-1 beta; IL-2, Interleukin-2; IL-4, Interleukin-4; IL-6, Interleukin-6; IL-8, Interleukin-8; IL-10; interleukin-10; K, potassium; L, lymphocytes; LUC, large unstained cells; MCH, mean cell hemoglobin; MCHC, mean cell hemoglobin concentration; MCP-1, Monocyte Chemoattractant Protein 1; MCV, mean cell volume; M, monocytes; N, neutrophils; Na, sodium; Phos, inorganic phosphorus; Plt, platelet count; RBC, erythrocyte count; RDW, red cell distribution width; Retic, absolute reticulocyte count; TNF-α Tumor-Necrosis Factor alpha; Total prot, total protein; Trig, triglycerides WBC; total leucocyte counts.
