# Supplementary material for: Intrastriatal Administration of AAV5-miHTT in Non-Human Primates and Rats Is Well Tolerated and Results in miHTT Transgene Expression in Key Areas of Huntington Disease Pathology
Source: Brain Sci. 2021 Jan 20;11(2):129. doi: 10.3390/brainsci11020129 (PMC7908995; doi:10.3390/brainsci11020129)
Supplement: Supplementary file 1 [file brainsci-11-00129-s001.zip › brainsci-1035997-Sup/Table S2.pdf]

Supplementary table 2: mi*HTT* expression in off-target organs a) NHP and b) rat

a)

Dose, gc/brain	miHTT expression in off-target organs in NHP (gc/µg of RNA)						
	**Adrenal gland**	**Cervical lymph node**	**Heart**	**Kidney**	**Liver**	**Spleen**	**Ovaries**
$2x10^{12}$	n=6 <LLOQ	n=6 <LLOQ	n=6 <LLOQ	n=5 $4.04x10^3$ ($9.98x10^2$)	n=6 <LLOQ	n=6 <LLOQ	Not analyzed
$7x10^{12}$	n=2 $4.73x10^3$ ($2.48x10^3$)	n=6 <LLOQ	n=1 $5.11x10^3$	n=1 $3.34x10^3$	n=2 $4.79x10^3$ ($1.51x10^3$)	n=6 <LLOQ	Not analyzed
$2x10^{13}$	n=3 $6.12x10^3$ ($3.54x10^3$)	n=6 <LLOQ	n=1 $4.22x10^3$	n=6 <LLOQ	n=4 $5.57x10^3$ ($1.38x10^3$)	n=6 <LLOQ	n=3 $2.35x10^4$ ($2.22x10^4$)

Data are mean (standard deviation). N=6 for all tissues except ovaries (N=3). Where lower, n represents positively determined concentration of vector DNA. LLOQ was 25 copies/reaction. gc, genome copies; LLOQ, lower limit of quantification; mi*HTT*, micro RNA targeting *HTT*; NHP, non-human primates.

b)

Time, months post dose	miHTT expression in off-target organs in rats (gc/µg of RNA)					
	**Adrenal gland**	**Cervical lymph node**	**Liver**	**Lung**	**Spinal cord**	**Spleen**
1	n=6 $1.80x10^4$ ($1.14x10^4$)	n=1 $2.86x10^3$	n=10 <LLOQ	n=10 <LLOQ	n=10 <LLOQ	n=10 <LLOQ
6	n=7 $2.63x10^4$ ($3.30x10^4$)	n=10 <LLOQ	n=10 <LLOQ	n=2 $3.98x10^3$ ($6.15x10^2$)	n=5 $4.81x10^3$ ($1.43x10^3$)	n=2 $5.54x10^3$ ($3.93x10^3$)

Data are mean (standard deviation). N=10 for all tissues. Where lower, n represents positively determined concentration of vector DNA. LLOQ was $2.5x10^3$ copies/µg of RNA. gc, genome copies; LLOQ, lower limit of quantification; mi*HTT*, micro RNA targeting *HTT*.
