# Supplementary material for: Intrastriatal Administration of AAV5-miHTT in Non-Human Primates and Rats Is Well Tolerated and Results in miHTT Transgene Expression in Key Areas of Huntington Disease Pathology
Source: Brain Sci. 2021 Jan 20;11(2):129. doi: 10.3390/brainsci11020129 (PMC7908995; doi:10.3390/brainsci11020129)
Supplement: Supplementary file 1 [file brainsci-11-00129-s001.zip › brainsci-1035997-Sup/Table S3.pdf]

Supplementary table 3. Clinical observations following AAV5-miHTT treatment in NHP or rats

**Clinical parameter**	**Cynomolgus monkeys (*Macaca fascicularis*)**	**Crl:CD(SD) Sprague Dawley rats**
Neurological assessment	Transient absence of reflex responses at an incidence similar to controls	Not reported
Ophthalmic assessment	No treatment effect	Not reported
Body weight	No treatment effect	Differences from controls were of small magnitude and within normal ranges
Food consumption	No treatment effect	No treatment effect
Water consumption	No treatment effect	No treatment effect
Body temperature	No treatment effect	No treatment effect
Peripheral Blood hematology	No treatment effect	No treatment effect
Blood Chemistry	No treatment effect	Differences considered unlikely to be treatment related
Organ weight	No treatment effect	No treatment effect
Macropathology	No treatment effect	No treatment-related lesions
