# Supplementary material for: Intrastriatal Administration of AAV5-miHTT in Non-Human Primates and Rats Is Well Tolerated and Results in miHTT Transgene Expression in Key Areas of Huntington Disease Pathology
Source: Brain Sci. 2021 Jan 20;11(2):129. doi: 10.3390/brainsci11020129 (PMC7908995; doi:10.3390/brainsci11020129)
Supplement: Supplementary file 1 [file brainsci-11-00129-s001.zip › brainsci-1035997-Sup/Table S4.pdf]

Supplementary table 4. Microscopic examination of the brain show astrocytes and microglial cell increases that are associated with the needle tract in rats 1 month after injection with vehicle or AAV5-miHTT. Data are number of male/female rats with minimal or slight increases in astrocytes or microglial cells associated with the needle tract at 1 month out of a total of 16 animals (eight male and eight female rats in each group).

**Cell type**	**Controls N=16**	**4×10^{9} N=16**	**4×10^{10} N=16**	**4×10^{11} N=16**
	**Number of male/female rats with increases**			
Astrocytes	4/6	6/6	5/5	6/4
Microglial cells	4/6	6/6	6/5	1/0
