# Supplementary figures and images for: Intrastriatal Administration of AAV5-miHTT in Non-Human Primates and Rats Is Well Tolerated and Results in miHTT Transgene Expression in Key Areas of Huntington Disease Pathology
Source: Brain Sci. 2021 Jan 20;11(2):129. doi: 10.3390/brainsci11020129 (PMC7908995; doi:10.3390/brainsci11020129)

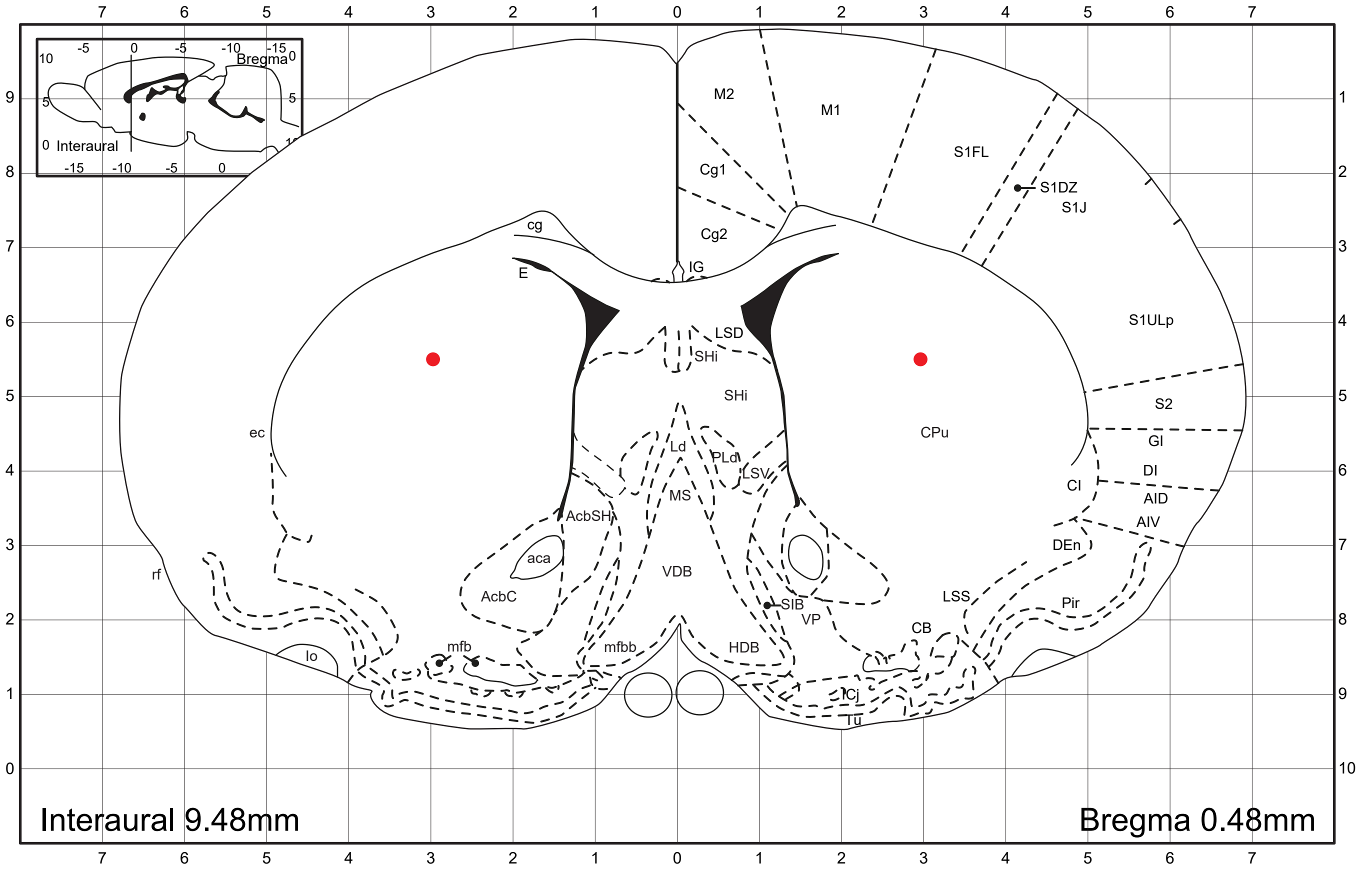

Supplement: Supplementary file 1 [file brainsci-11-00129-s001.zip › brainsci-1035997-Sup/Spronck Brain Sciences Fig S1_JAN 10.pdf]

**A**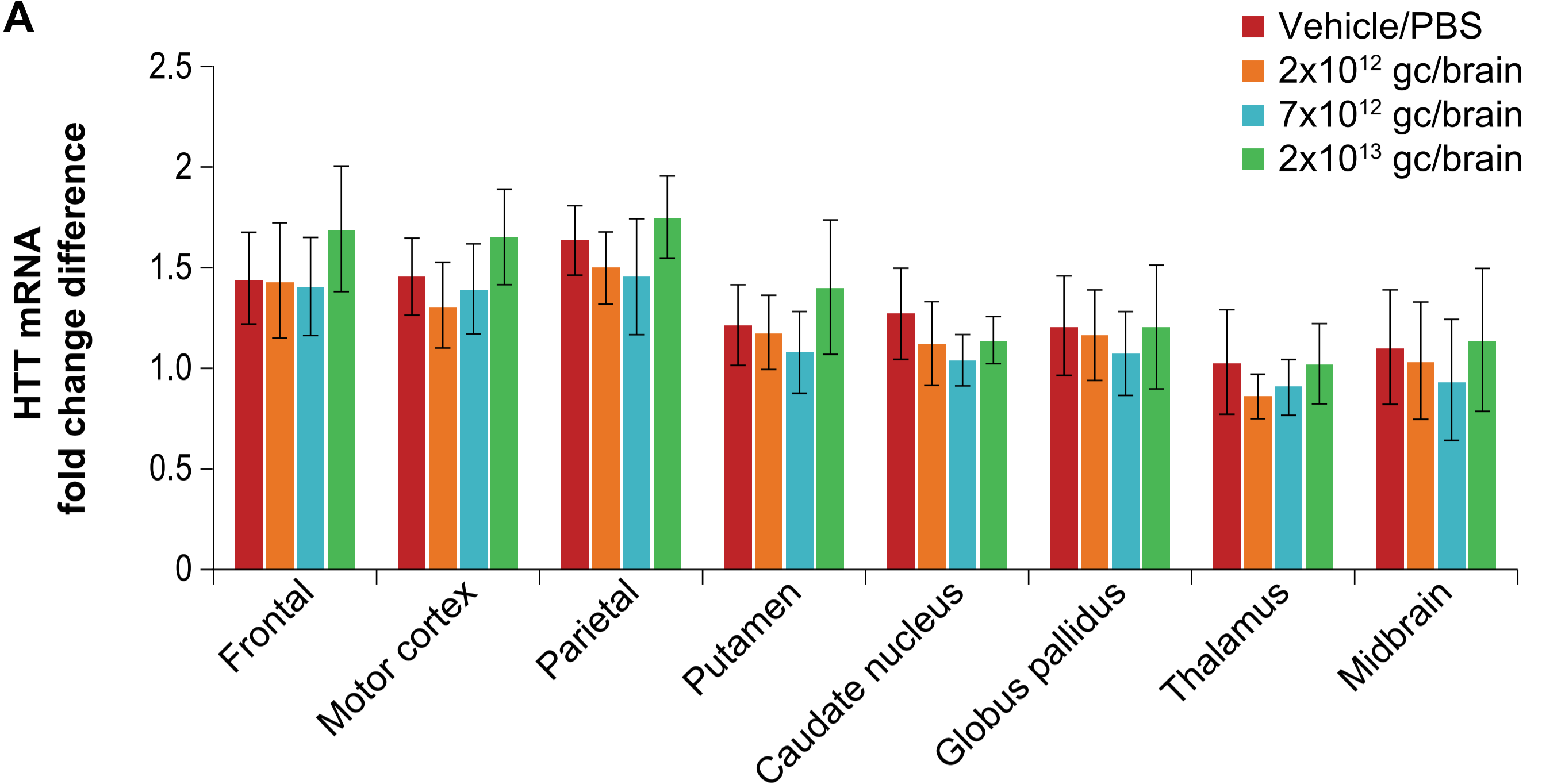**B**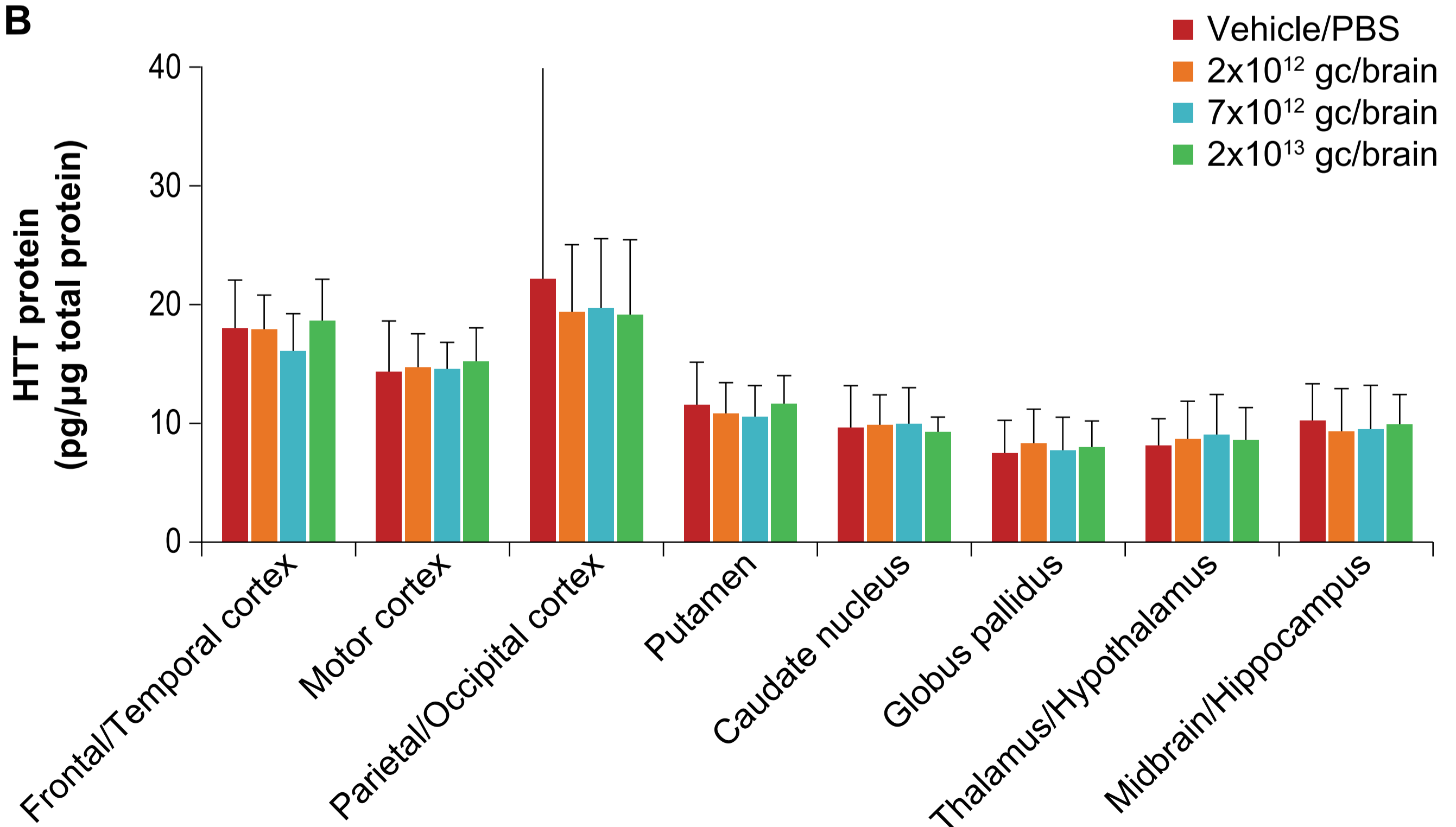

Supplementary figure 2. HTT mRNA gene expression (a) and HTT protein levels in the brain of NHP (b)

Supplement: Supplementary file 1 [file brainsci-11-00129-s001.zip › brainsci-1035997-Sup/Spronck Brain Sciences Fig S3_JAN 10.pdf]

### Plasma vector DNA

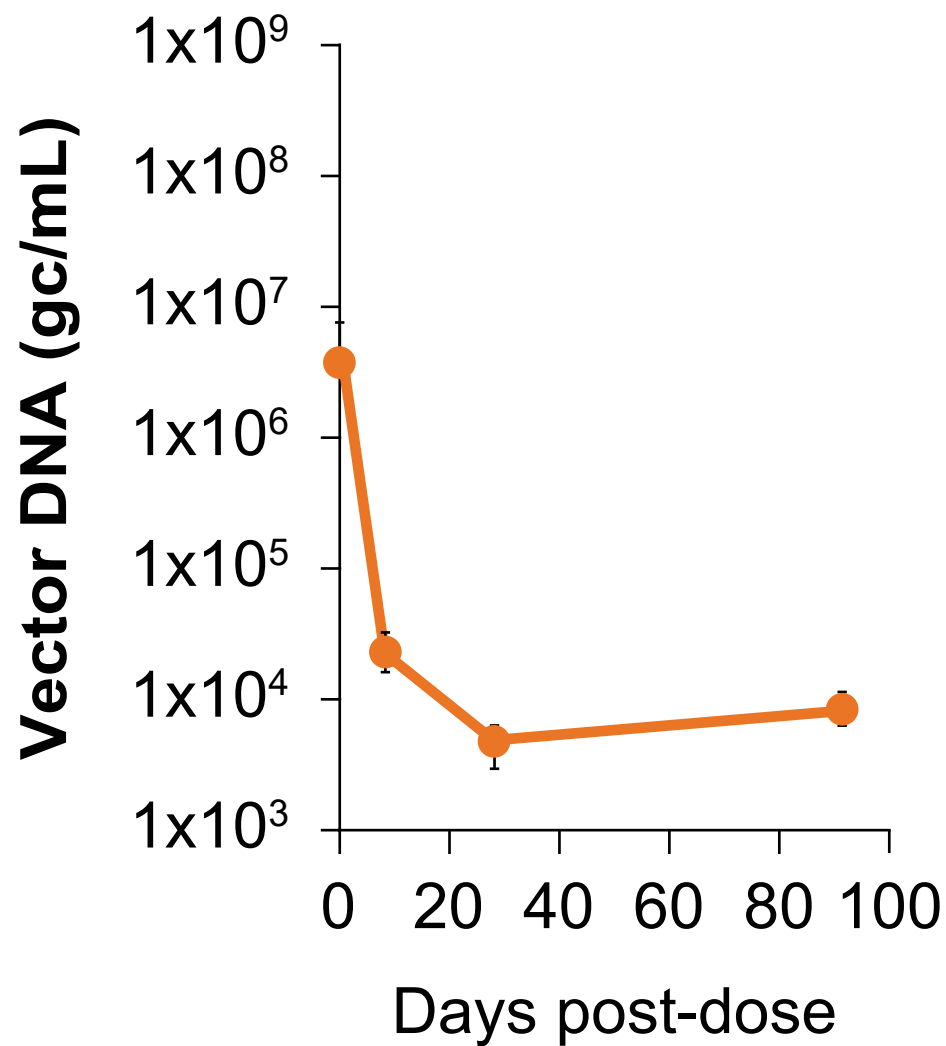

### Urine vector DNA

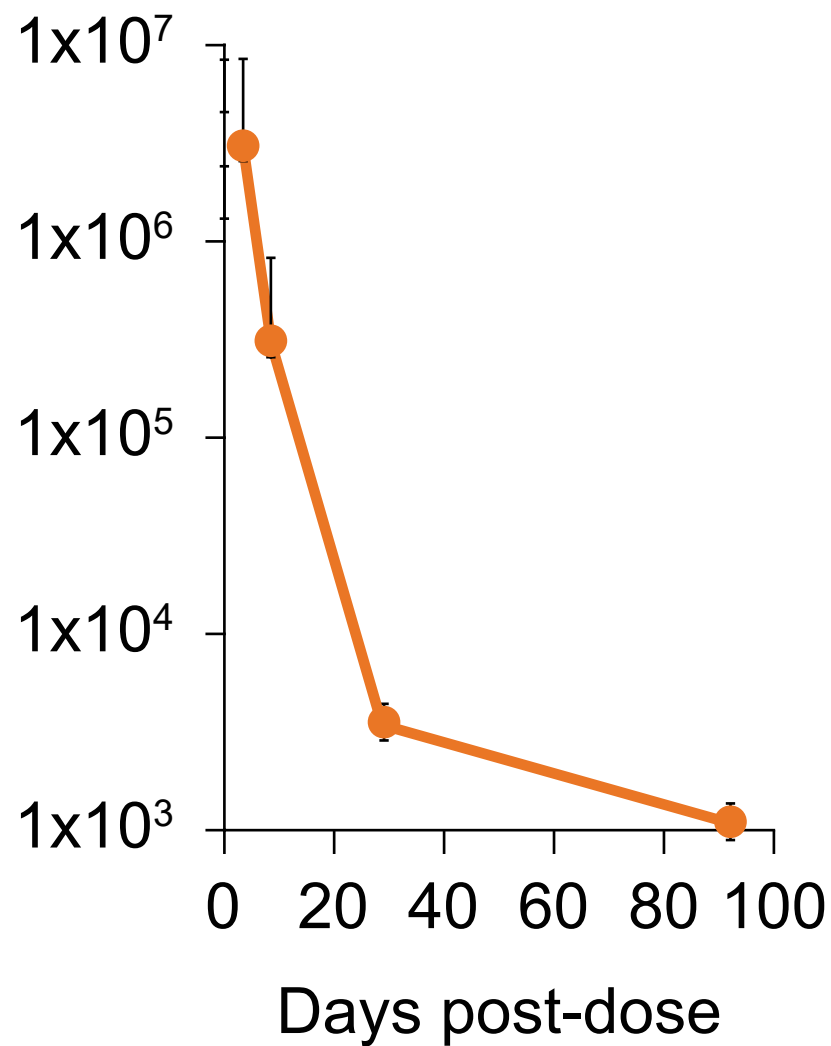

### Saliva vector DNA

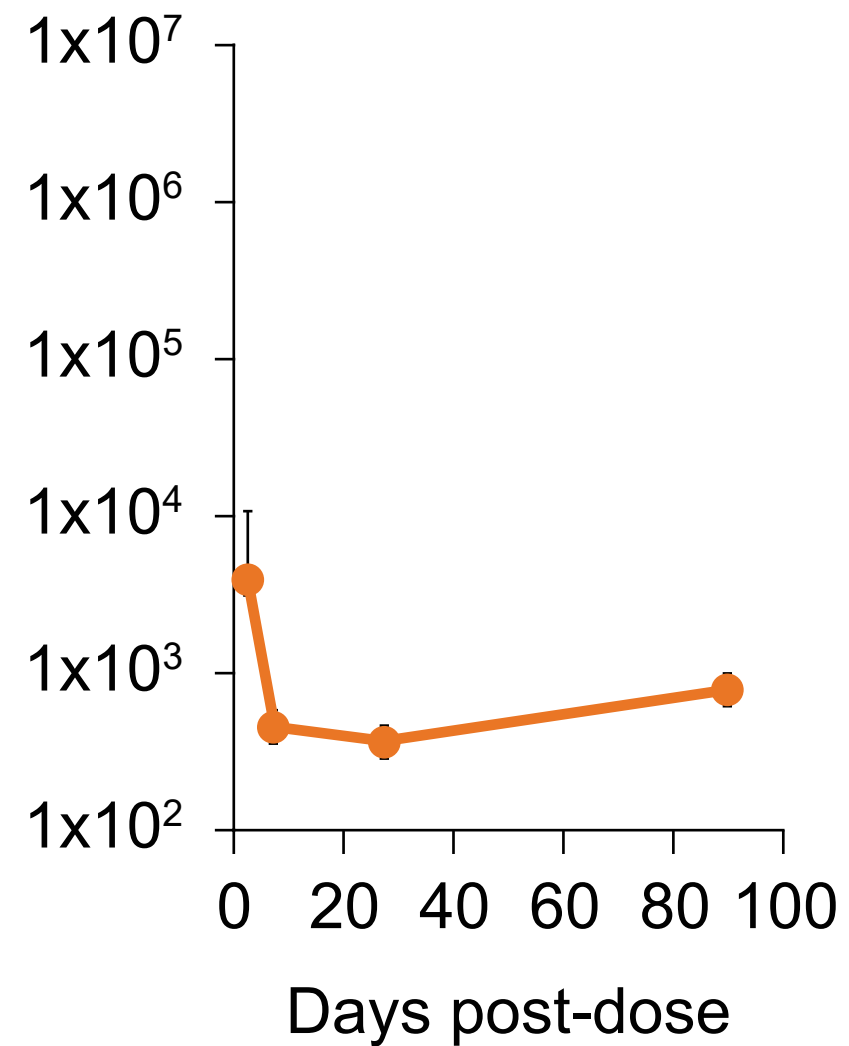

Supplement: Supplementary file 1 [file brainsci-11-00129-s001.zip › brainsci-1035997-Sup/Spronck Brain Sciences Fig S4_JAN 10.pdf]
